# Series of 10 dengue fever cases with unusual presentations and complications in Sri Lanka: a single centre experience in 2016

**DOI:** 10.1186/s12879-018-3596-5

**Published:** 2018-12-18

**Authors:** S. A. M. Kularatne, Udaya Ralapanawa, Chamara Dalugama, Jayanika Jayasinghe, Sawandika Rupasinghe, Prabashini Kumarihamy

**Affiliations:** 1 Department of Medicine, Faculty of Medicine, University of Peradeniya, Peradeniya, Sri Lanka; 2 Teaching Hospital Peradeniya, Peradeniya, Sri Lanka

**Keywords:** Dengue, Expanded dengue syndrome, Fulminant liver failure, Myocarditis, Septic shock, Dengue shock syndrome, DHF, Sri Lanka

## Abstract

**Background:**

Dengue has global importance as a dreaded arboviral infection. It has 4 serotypes of epidemiological imporatnce. The classification denotes two clinical spectrums- dengue fever (DF) and dengue haemorragic fever (DHF). Most cases are stereotype and amenable to fluid resuscitation. However, unusual manifestations cause fatalities and often overlooked. This study describes 10 such dengue cases to fill the knowledge gaps.

**Case presentation:**

All 10 patients presented to the Teaching Hospital, Peradeniya, Sri Lanka during mid-year epidemic of dengue in 2016. The mean age is 27 years (range 12-51 years) comprising 6 females and 4 males. The group had 7 DHF, 3 DF and 2 primary dengue infections who predominantly had severe bleeding into gut. Other potentially life threatening problems were acute severe hepatitis, severe septic shock, myocarditis, erratic rapid plasma leak, intracranial bleeding, diarrhoea and decompenstaed dengue shock due to 3rd space fluid leak. Blood transfusions and other empirical therapeutic methods were used apart from meticulous fluid management to suit issues of each patient. Bedside ultrasound scanning helped early detection of critical phase. All recovered fully.

**Conclusions:**

Dengue is an extremely challenging infection to treat in the globe today. Above unusual presentation and complications could be fatal, if not detected early where therapeutic window period is very short. Clinicians need awareness of these problems which are not uncommon, but underreported and often overlooked. The clinical management of each patient was described for the purpose sharing the experiences.

## Background

Dengue is the most common arboviral infection in the Southeast Asia. Dengue virus has four related but antigenically distinct serotypes: DENV-1, DENV-2, DENV-3, and DENV-4 [1]. The global burden of dengue has increased in recent decades causing huge impact on both human health and the national economics [1–3] . Dengue infection has a diverse clinical presentation ranging from asymptomatic subclinical infection to severe multi-organ involvement [3]. Although, vascular plasma leak is the commonest manifestation, dengue can manifest in multitude of unusual presentations due to organ dysfunction that can carry high mortality [2, 3]. Early detection of such manifestations and prompt action could avert the adverse outcome where clinicians need knowledge and experience. Aim of this case series is to present 10 such unusual dengue cases managed in a single hospital over 1 year period. These patients presented to Teaching Hospital, Peradeniya (THP), Sri Lanka in 2016 and recovered fully following problem based tailored management.

### Case 1: (erratic rapid plasma leak during early critical phase)

A 22-year-old female admitted to THP with a one-day history of fever proceeded by frontal headache of 3 days. On admission, she had arthralgia, myalgia, mild postural dizziness and nausea. She has passed urine normal amount. She was hemodynamically stable with a blood pressure of 96/64 mmHg without a postural drop. Abdomen was soft and non-tender. Clinically, she did not have evidence of plasma leak. Her blood test was positive for dengue NS1 antigen. On 3rd day of fever, ultra sound scan of abdomen detected thin rim of free fluid in the hepato-renal pouch and moderate gall bladder wall oedema with mild pericholycystic fluid. She did not have pleural effusion or ascites. Neither she had postural drop of blood pressure, tachycardia or right hypochondrial tenderness. However, her haematocrit has risen from 33 to 38%. In a flash, within the next 6 h, she developed significant ascites (moderate) and bilateral moderate pleural effusions with a reduction of urine output. She had fluctuation of urine output and blood pressure and required several normal saline boluses and Dextran-40 along with frusemide to maintain vital parameters. Sixty percent of her calculated fluid quota was utilized in the 1st 12 h of tentative critical phase. Her clinical status gradually improved within the next 3 days. But, there was delayed resolution of effusion and ascites. Her serum albumin level dropped during the critical phase and took days reverse. Her recovery was uneventful and discharged on day 6 of the hospital stay. She had erratic rapid leaking of plasma into serous cavities during critical phase.

### Case 2: (severe hepatitis with increased transaminases and gross ascites after critical phase)

A previously healthy 39-year-old female, admitted to the THP with a history of fever for 4 days. She had nausea, vomiting, arthralgia, myalgia and headache. She did not have any bleeding manifestations or abdominal pain. On examination, she had mild dehydration with low volume pulse. Blood pressure was 100/80 mmHg in supine position and 90/80 mmHg on standing. Right lung base was stony dull on percussion and had absent breath sounds. Ultrasound scan revealed a right sided plural effusion with free fluid in the abdomen. The patient was managed as critical phase of dengue haemorrhagic fever (DHF) with meticulous titration of fluids according to the haematocrit values. She remained hemodynamically stable with a stable haematocrit values during the critical phase. On day 7 of illness, dengue serology showed positive IgM and IgG titers. After completion of critical phase on 7th day of the illness, she complained of abdominal pain and back pain. Clinical examination found s mild icterus and tense ascites. Laboratory investigations revealed a marked rise in liver enzyme levels (ALT 204 to 1391 u/L and AST 505 to 4519 u/L) with an INR of 1.9. Diagnosis of acute hepatitis leading into acute liver failure was made and viral hepatitis was excluded by doing hepatitis A IgM, hepatitis B surface antigen and hepatitis C IgM which were negative. She denied self-medication with high doses of paracetamol. Further, she was treated with intravenous N acetyl cysteine 150 mg/hour infusion as an empirical treatment. Her low albumin level was corrected with intravenous human albumin administration. Antibiotics including oral metronidazole and intravenous ceftriaxone was administered at the same time to cover bacterial infections. She was given intravenous vitamin K for 3 days to prevent clotting factor depletion whilst monitoring liver transaminases and clotting parameters. Finally she was discharged on 12th day of the illness with near normal liver transaminases and normal clotting profile without residual free fluids in her abdomen. Further follow of after 21 days revealed completely normal liver biochemistry.

### Case 3: (DEN 2, intracranial Haemorrhage in DHF)

A19- year-old male, previously healthy university student admitted to THP having a febrile illness with arthralgia and myalgia for 5 days duration. On the way to the hospital, the patient had postural dizziness and fainting attack causing impact on the forehead. Soon after admission, he developed a generalized tonic-clonic seizure which lasted for 5 min with post ictal drowsiness. On examination, he was not pale but had conjunctival hemorrhages. He had a contusion over the forehead due to fall. He was hemodynamically stable with a blood pressure of 126/90 mmHg and a pulse rate of 92 beats per minute without clinical evidence of plasma leaking. Ultrasound scan revealed a thin rim of free fluid in the abdomen. Dengue NS 1 antigen and Dengue Ig M and IgG both were positive. Serotype of dengue was identified as DEN 2. Diagnosis of DHF entering into critical phase was made and commenced intense monitoring with administration of intravenous and oral fluid according to guidelines, Meanwhile, the patient was found to be drowsy but arousable without having any lateralizing neurological deficits. Both optic fundi were normal. Non-contrast CT brain revealed bilateral frontal lobe hyperdense areas with mild cerebral oedema with minimal midline shift, suggestive of intra-cranial hemorrhages. His clotting parameters were within the normal limits. He was transfused with platelets to keep the platelet count more than 50 × 10^6^/L and managed conservatively with adequate intravenous fluids, intravenous antibiotics and antiepileptic drugs. He was started on intravenous phenytoin sodium and later converted to oral phenytoin. Cerebral oedema was managed with intravenous dexamethasone and intravenous mannitol. He was administered with intravenous tranexamic acid to retard further bleeding. Critical phase was uneventful. His headache and drowsiness improved over the next 5 days and discharged with oral antiepileptics.

### Case 4: (DEN 1 causing myocarditis and DHF together)

A-17-year-old previously healthy female presented to THP with a history of fever for 2 days associated with body aches and nausea. She didn’t have any abdominal pain, bleeding manifestations or postural symptoms. On examination, she was flushed and febrile but was not pale or icteric. She was mildly dehydrated. Blood pressure was 100/70 mmHg, pulse rate 100 beats/min and capillary refilling time (CRFT) was less than 2 s. On abdominal examination, there was no free fluid. Lung fields were clear on respiratory system examination. Other systems examination was normal.

Her NS1 antigen was positive and serotype was identified as DEN1. She was managed as dengue fever with continuous monitoring. On the 3rd day of fever, she complained of retrosternal chest pain and undue tiredness. At that time her cardiovascular system examination was normal and electrocardiogram (ECG) showed acute T wave inversion in V2-V5 leads. Troponin I was negative and 2D echo showed global left ventricular hypokinesia and mild impairment of LV function. Ejection fraction was 40–45%. She was treated as having dengue fever complicated by myocarditis. Intravenous hydrocortisone 200 mg 8 hourly was administered for 2 days to reduce myocardial inflammation. On the 4rd day following admission, she complained of abdominal pain and ultrasound scanning revealed free fluid in hepato-renal pouch. Blood pressure was 100/70 mmHg, pulse rate 70 bpm, and CRFT was less than 2 s. She was taken to High Dependency Unit (HDU) and was managed as having DHF complicated with myocarditis with continuous monitoring and with careful administration of fluid to avoid fluid overload. She was discharged on day 7 of illness after recovering from critical phase of dengue fever. She was advised on limiting physical activities. During the follow up on day 14 of the illness, ECG showed reversal of T inversions. Echocardiogram showed improvement of left ventricular function with an ejection fraction of 55%.

### Case 5: (acute GI bleeding and hepatitis in DF)

A-22-year old previously healthy male admitted to THP with a history of on and off fever for 10 days associated with 3 bouts of hematemesis and melaena. On examination, his pulse rate was 88 beats/m, blood pressure was 90/60 mmHg and CRFT was less than 2 s and lungs were normal. Abdomen was soft and there was no detectable free fluid. Rest of the examination was unremakable. Serology for dengue IgM and IgG were positive on admission. His liver enzymes were high on admission (AST 840 U/L and ALT 560 U/L) with a high INR value of 2.1. His complete blood count showed 11.5 g/dl of haemoglobin and platelet count of 144 × 10^9^/l. Ultrasound examination of abdomen did not show any evidence of leaking thus, DHF was excluded. Hence, the patient was managed as in primary dengue fever with bleeding manifestations. Intravenous fluids were given along with tranexamic acid and vitamin K to reduce bleeding. Intravenous infusion of omeprazole was continued for 24 h and converted to twice day intravenous boluses. He was started in intravenous N acetyl cysteine infusion as liver transaminases were high. His symptoms resolved within the next few days, with symptomatic management.

### Case 6: (DEN 2 primary infection and bleeding into GIT and GUT)

A-12-year old previously well female child was transferred to THP from a private hospital due to fever for 5 days associated with melena, haematemesis and haematuria with passage of blood clots. She did not have abdominal pain or any other warning signs of dengue on admission.

On examination, she was ill looking, adequately hydrated and GCS was 15/15. Blood pressure was 125/75 mmHg, pulse rate was 90 beats per minute and capillary refilling time was less than 2 s. On respiratory examination lungs were clear and on abdominal examination the abdomen was soft and non tender. Rest of the clinical examination was normal. Both NS1 and IgM were positive and dengue PCR revealed serotype of DEN 2. Ultrasound examination of abdomen did not show any evidence of plasma leaking. She was managed as having primary dengue fever with bleeding manifestations. Her liver enzymes were only mildly elevated (AST 87 u/L and ALT 56 u/L) with a normal clotting profile. Complete blood count revealed hemoglobin of 7 g/dl and platelet count of 17 × 10^9^/μL. Due to low haemoglobin, she was transfused with 1 pint of blood and 4 units of platelets. Her symptoms resolved within the next few days.

### Case 7: (DEN 2, severe diarrhoea, DHF, profound shock, sepsis and occult bleeding, need of massive transfusion)

A 14-year-old boy presented to THP with a history of fever for 2 days along with headache, arthralgia and myalgia. He did not complain of abdominal pain and did not have bleeding manifestations or any other warning symptoms. On examination, blood pressure was 110/70 mmHg and pulse rate was 100 beat per minute and capillary refilling time was less than 2 s. The abdomen was soft and non-tender and there was no free fluid. Lung fields were clear on respiratory system examination. Rest of the examination was normal. His NS-1 was positive and the PCR appeared as DEN 2 serotype. The patient was managed as having dengue fever. He continued to have fever spikes for 4 days following admission. On the 5th day following admission, he developed postural dizziness, vomiting and heavy diarrhoea. On examination, he was febrile, dehydrated, flushed and had warm peripheries. Blood pressure was 90/60 mmHg, pulse rate was 130 beats per minute and a capillary refilling time of 2 s. Ultrasound examination of abdomen revealed free fluid in the hepato-renal pouch with increased gall bladder wall thickness. He was clinically diagnosed as having DHF complicated with septic shock and gastroenteritis. He was taken to HDU and critical phase monitoring commenced. His c-reactive protein was high 112 mg/dl. Broad-spectrum intravenous antibiotics (ceftriaxone and metronidazole) were started cover the sepsis after taking blood and urine cultures. Within about 1 h, the patient deteriorated significantly and continued to have vomiting and diarrhoea. Blood pressure dropped to 60/30 mmHg and the pulse rate increased to 120 beats/min. Several fluid boluses were given including normal saline and IV Dextran 40. The haematocrit value dropped from 36 to 30 and patient went into decompensated shock with no urine output. He needed continuous transfusion of whole blood amounting to 9 pints over 20 h to maintain blood pressure and urine output. However, there were no obvious bleeding sites. Further, intravenous noradrenaline infusion supported the blood pressure. Gradually patient improved with fluid, blood, antibiotics and vasopressors. He was given intravenous antibiotics for total of 7 days. Vasopressor was gradually weaned off. He was plethoric during convalescence due to over transfusion and was discharged on day 8 of admission.

### Case 8 (presenting as dysentery and in compensated shock in DHF)

A previously well 36-year-old Buddhist monk presented to THP with a history of a febrile illness with generalized malaise for 4 days duration. His main complaint was vomiting and diarrhea of same duration. He did not have any postural symptoms, bleeding manifestations or abdominal pain at presentation. On examination, he was febrile and was not pale or icteric. Blood pressure was 120/100 mmHg with a pulse rate of 110 beats per minute and capillary refilling time of 2 s. On respiratory system examination, there was bilateral plural effusion and on examination of the abdomen there was shifting dullness. Other systems examination was normal. Ultrasound examination of abdomen revealed moderate amount of free fluid in the abdomen. Blood and urine were taken for investigations. His NS 1 antigen was positive, and serotype was identified as DEN 2. The patient was immediately taken to HDU and was managed as compensated shock of dengue hemorrhagic fever. Initial investigations revealed a platelet count of 15 × 10^9^/l, and haematocrit of 57%. With meticulous fluid management he recovered. Thus, this patient had clinical picture of dysentery associated with DHF presenting at the peak of critical phase.

### Case 9: (occult leaking of plasma leading to undetected decompensated shock)

A 51-one-year-old previously healthy female admitted with a history a febrile illness with arthralgia and myalgia for 4 days. Her NS1 antigen was positive on admission. She was ill and complained of postural dizziness and abdominal pain. On examination, she was ill looking, dehydrated and had bluish cold peripheries. She had central cyanosis and collapsed superficial veins. Her supine blood pressure was recorded as 90/80 mmHg and standing blood pressure was unable to measure due to severe postural symptoms. Capillary refilling time was prolonged, and her respiratory rate was 24 breaths per minute. Lungs were clear and clinically there was no evidence of free fluid in abdomen and pleura. She did not pass urine for 12 h. She was clinically diagnosed to have dengue haemorrhagic fever with decompensated shock. Then she was admitted to the HDU and critical phase management was started. Ultrasound scan of the abdomen did not show free fluid in peritoneal cavity despite patient was possibly in the peak of plasma leaking. However, 12 h after admission, repeat ultrasound scan showed thin rim of free fluid in the hepatorenal pouch. She was resuscitated with boluses of crystalloids and colloids., She became hemodynamically stable gradually and took about 8 h to gain warm peripheries. Fluid management and monitoring was continued, and her symptoms improved within the next 2 days. Although she went in to decompensated shock due to DHF, she had minimum detectable amount free fluid in the abdomen in the later phase of leaking.

### Case 10: (DF complicating severe septic shock)

A 34-year-old female presented with a febrile illness with arthralgia and myalgia for 2 days duration. Her Dengue NS1 was positive. Her hemodynamic parameters were stable on admission. She was having continuous fever on day 6 of illness. There was no evidence of hemoconcentration or plasma leak and managed as uncomplicated dengue fever. She was kept on intravenous saline infusion at a slower rate. On 6th day of fever she developed cough and shortness of breath. Auscultation of lungs heard crepitations in bases. Over next 6 h she was not improving despite continuous infusion of normal saline and commencing antibiotics. Later, she became agitated and restless and was confused. She had warm peripheries despite blood pressure of 80/40 mmHg which further dropped to 60/30 mmHg. She had pulse rate of 108 beats/ min. There were widespread coarse crackles in the both lung fields involving all 3 zones. Her oxygen saturation dropped to 85% on room air. Her haematocrit remained within normal range. To counter the shock, she was given more intravenous normal saline, Dextran 40 and 2 units of blood transfusion. Then, she developed pulmonary oedema and required CPAP in the intensive care unit with high flow oxygen and intravenous frusemide. Patient was treated with intravenous meropenum 1 g 8 hourly and metronidazole. She had very high CRP and procalcitonin levels suggestive of severe sepsis. After 6 h of resuscitation her blood pressure got stabilized and she recovered completely over next 5 days. She was diagnosed as dengue fever complicated by septic shock probably originating from lungs even though, dengue shock syndrome (DSS) was contemplated at the outset.

## Discussion and conclusion

Our case series compiles summaries of 10 confirmed dengue cases with wide array of unusual manifestation which are potentially fatal, in a single centre (THP) in the central hills of Sri Lanka. All these patients presented during mid-year outbreak of dengue in 2016 when serotype transition occurring from DEN 1 to DEN 2 that finally led to a massive outbreak of DEN 2 in 2017 in Sri Lanka. In these cases, females (n,6) out number males (n,4) and 7 patient had DHF. Out of 3 patients who had DF, 2 developed severe GI bleeding while other one developed severe septic shock that was mistaken for dengue shock syndrome (DSS) initially. Other unusual manifestations highlighted are hepatic dysfunction, myocarditis, erratic plama leak, ICH, occult blood loss, decompensated shock etc. Early detection of these manifestation and taking appropriate clinical decisions such as blood transfusions, antibiotics, and other empirical treatments saved all lives.

Most such manifestations of dengue infection are underreported, under recognized or not casually linked to dengue fever. Therefore, vigilance and anticipation are needed in managing dengue beyond the most common stable type of plasma leak in DHF.

Common life threatening complications related to DF and DHF include hepatic dysfunction leading to acute fulminant hepatic failure [3], musculoskeletal complications such as myositis and rhabdomyolysis [4], acute renal failure [5], cardiac complications such as myocarditis [6], life threatening bleeding such as gastrointestinal and intracranial bleeding [7], endocrine complications such as precipitating diabetic ketoacidosis [8] and neurological complications such as Guillain Barre syndrome and encephalopathy [9]. Early identification and early approach for appropriate management strategies are important to reduce morbidity and mortality of such cases. Better understanding of the disease dynamics has improved the outcome over time but still timely diagnosis and management is a challenge.

This case series comprises primary dengue infection, dengue fever (DF) and dengue hemorrhagic fever (DHF) all associated with unusual manifestaions. Importantly some life-threatening complications were observed in both primary dengue infection and DF without leaking. Patients in cases 1,2,3,4,7,8 and 9 developed DHF whereas patients in cases 5, 6 and 10 had DF. Some presented with bleeding manifestations while the others developed complications mentioned above.

Dengue can present with a diverse clinical spectrum ranging from asymptomatic infection or simple undifferentiated fever to DHF with multiorgan failure. Pathological hallmark of dengue hemorrhagic fever is increased capillary permeability with extravasation of fluids during the critical phase of dengue fever [3, 10]. The onset of critical phase is determined by clinically or radiologically demonstrable pleura effusion or ascites and/or evidence of hemoconcentration as shown by increased haematocrit in serial measurements [3, 11]. The critical phase lasts for a period of 24–48 h in which rate of plasma leak gradually peaks and comes down to the baseline. But this typical pattern is not appreciated all the time. Case 1 describes a young erratic leaker. His plasma leak peaked within 1st 12 h of critical phase evidenced by rapidly rising haematocrit and rapidly developing pleural effusions and ascites necessitating use of more than 60% of fluid quota within first 12 h. This type erratic leaker needs to be identified early with frequent monitoring of clinical parameters and haematocrit and fluid need to be titrated accordingly. The same patient had obvious fluid leak into peritoneal cavity and pleural spaces with hypoalbunaemia and took time for reabsorption. Case 9 describes a female who presented with decompensated shock. She had evidence of hemoconcentration with a high haematocrit. But on presentation clinically and ultrasonically she had no objective evidence of plasma leak into serous cavities. This highlights that although plasma leak is describes as selective to pleural, pericardial and peritoneal cavities, there can be substantial amount of fluid leaking in to 3rd space of unknown sites. Absence of objective fluid leak should not delay the treating physician making a diagnosis of DHF in the presence of evidence of intravascular volume depletion and hemoconcentration. However, frequent use of ultrasound examination has enhanced early detection of plasma leak. In THP, ultrasound scanners are available in the wards where dengue patients are treated to do bedside scaning without mobilizing patients to scanning rooms.

Liver dysfunction is a well-recognized feature in both dengue fever and DHF. Patients with dengue fever complaining of abdominal pain, nausea, vomiting and anorexia should alert the physician of the possibility of liver involvement [12]. Aetio-pathogenesis in liver dysfunction in dengue fever is yet to be elucidated. Direct effects of the virus or host immune response on liver cells, circulatory compromise, metabolic acidosis and/or hypoxia caused by hypotension or localized vascular leakage inside the liver are possible mechanisms postulated to explain the liver dysfunction [13, 14]. Case 2 describes patient with DHF developing acute liver failure and case 5 describes a patient with DF without leaking developing liver dysfunction, coagulopathy and gastrointestinal bleeding. Both cases were successfully managed with adequately maintaining the hydration status and intravenous N acetyl cysteine. NAC scavenges free radicals, improves antioxidant defense and acts as a vasodilator to improve oxygen delivery and consumption [15]. However, efficacy of NAC needs to be validated with further studies.

Bleeding manifestations are seen both in dengue fever and dengue hemorrhagic fever. Despite the name, cardinal feature that differentiate dengue fever from DHF is not hemorrhage but leaking. The underlying mechanisms responsible for bleeding in dengue infections remain poorly understood. Thrombocytopenia is universal in DHF and most of the patients with DF [16]. Platelet functions is abnormal in acute dengue fever mainly due to impaired ADP mediated platelet aggregation [17].Isarangkura et al. reported that platelet survival is less in acute dengue fever [18]. Mild prolongation of prothrombin time and activated partial thromboplastin times with reduction in fibrinogen levels were reported in some studies in patients without liver involvement [19, 20]. Patients with prolonged shock with multi-organ dysfunction and those with acute liver failure had major gastrointestinal bleeds contributed by deranged clotting and gut ischemia. Case 5 describes a patient with DF without leaking who had normal platelets with severe liver involvement leading to coagulopathy and gastrointestinal bleeding. In contrast in case 6, a primary DF patient developed severe gastrointestinal bleeding needing blood and platelet transfusions. Her liver enzymes were marginally elevated and had a normal clotting profile. Her main risk factor for a gastrointestinal bleed, haematuria was low platelet count.

Intracranial hemorrhage in dengue fever is a rare but a grave complication. Mechanism of intracranial bleeding is still not clearly described, it is postulated that it could be due to the interplay between coagulopathy, platelet dysfunction, thrombocytopenia, and vasculopathy [21, 22]. In our patient described in case 3 presented in the peak of leaking with postural symptoms. On admission, his platelet count was16 × 10^9^/μl. He had history of a fall with head impact and soon after admission patient sustained a generalized tonic clonic fit. Later he was found to have bilateral frontal lobe hemorrhages. This could be either traumatic following the fall or could be spontaneous which might have caused the fall. Management of an ICH in a dengue patient is controversial as the causative factors such as vasculopathy and platelet dysfunction are usually still present and irreversible while surgery is undertaken. No studies have been performed on place for surgery for ICH in dengue fever. Low platelets are the main risk factor for an ICH in dengue fever. There is no consensus on when to transfuse platelets and place for primary prophylaxis. Some studies have recommended prophylaxis platelet transfusions when the platelet count is very low [23, 24]. In 2011, Kurukularatne et al. strongly concluded that prophylactic platelet transfusion is associated with hazards and wastage without any hematological benefit and therefore, should not be adopted as a routine clinical practice [25]. Our patient received platelet transfusion as a secondary prophylactic measure as the platelet count was very low.

Dengue fever and DHF are associated with a wide spectrum cardiac complication. Kularatne et al. showed that 62.5% of 120 adults with dengue fever had an abnormal electrocardiogram [26]. Most cardiac complications are clinically mild and self-limiting, therefore, they are under diagnosed [27]. Myocardial involvement in dengue is yet to be fully described, but it can be due to direct viral invasion of the myocardium or cytokine mediated immune injury [28]. In case 4, young patient had evidence of myocarditis in ECG and left ventricular global hypokinesia in the 2D Echo. She was managed conservatively with meticulous fluid management and with a short course of steroids. Theoretically, steroids help to reduce inflammation of myocardium, thus improving contractility. Her follow up Echo in 2 weeks showed normalization of left ventricular systolic function.

Clinically considerable proportion of dengue patients who presented to the hospital can have bacterial co-infection [29]. Bacterial co-infection can be easily overlooked in the dengue epidemic setting. Delay in diagnosis and delay in anti-microbial therapy will have adverse outcome. Bacteremia in dengue fever is mainly Gram negative. It is probably caused by the breakdown of the intestinal mucosal barrier in severe dengue infection. In Case 7, the patient had DHF complicated with septic shock. The focus of sepsis is probably the gut, as he had diarrhoea and sepsis developed during leaking phase and gut ischemia probable led to breach in mucosal defense and gram-negative sepsis. Low blood pressure in tachycardia could have been easily overlooked attributing to dengue shock syndrome, but the disproportionately high pulse rate and warm peripheries in the background of shock alerted the treating physician of the possible underlying sepsis. Prompt use of antibiotics and judicious use of vasopressors were lifesaving. It is intriguing that he needed massive transfusions to maintain his blood pressure and save the life. This case provide empirical evidence for blood transfusion sever dengue infection Moreover, Case 10 describes a patient developed dengue fever and septic shock probably originating from the lung. Patient was treated with blood transfusion and intravenous crystalloids and colloids to overcome the shock which resulted in pulmonary edema. Judicious use of vasopressors is important in such instance to prevent volume overload. Supportive respiratory care over many hours maintained the oxygenation. The Case 8 describe presentation of dengue predominantly with diarrhea that might mislead the clinician as bacillary dysentery.

Lessons learnt from managing difficult cases of dengue as we presented here need sharing. Similar cases or situations may be happening at any given time in the globe as dengue is mostly seen everywhere. These clinical observations needs explanation on pathophysiological basis, but the knowledge about pathology and pathophysiology in dengue needs further improvement. Better treatment options are needed to improve the outcome of dengue.

## Abbreviations

ADP: Adenine di-Phosphate
ALT: Alanine transaminase
AST: Aspartate transaminase
CPAP: Continuous Positive Airway Pressure
CRFT: Capillary Refilling Time
DEN1: Dengue virus serotype 1
DHF: Dengue Haemorrhagic Fever
FBC: Full Blood Count
HDU: High Dependency Unit
ICH: Intra Cranial Haemorrhage
NAC: N-Acetyl Cysteine
PCR: Polymerase chain reaction
PCV: Packed Cell Volume
THP: Teaching Hospital Peradeniya
